# Cooking, Digestion, and In Vitro Colonic Fermentation of Nigerian Wholegrains Affect Phenolic Acid Metabolism and Gut Microbiota Composition

**DOI:** 10.3390/ijms241814111

**Published:** 2023-09-14

**Authors:** Oghenerukevwe Anne Obayiuwana, Volker Behrends, Yolanda Calle-Patino, Monica Barone, Silvia Turroni, Patrizia Brigidi, Adele Costabile, Giulia Corona

**Affiliations:** 1 School of Life and Health Sciences, University of Roehampton, London SW15 4JD, UK; anneobayiuwana@gmail.com (O.A.O.);; 2 Department of Medical Biochemistry, University of Benin, Benin 300283, Nigeria; 3 Microbiomics Unit, Department of Medical and Surgical Sciences, University of Bologna, 40138 Bologna, Italy; 4 Unit of Microbiome Science and Biotechnology, Department of Pharmacy and Biotechnology, University of Bologna, 40126 Bologna, Italy

**Keywords:** wholegrains, cooking, polyphenols, gut microbiota, digestion, fermentation, bioaccessibility, next-generation sequencing, UPLC-MS/MS

## Abstract

Wholegrains contain both fibre and phenolic acids (PAs), and their gastrointestinal modifications are critical for their bioavailability and bioactivity. We evaluated the modifications on the PA profile and gut microbiota composition of selected Nigerian wholegrains, following cooking and gastrointestinal digestion. Red fonio, red millet, red sorghum, and white corn were cooked, digested, and fermented using an in vitro colonic model. A total of 26 PA derivatives were quantified in soluble and bound fractions using Ultraperformance Liquid Chromatography-Tandem Mass Spectrometry (UPLC-MS/MS) analysis. DNA samples were analysed using 16S rRNA amplicon sequencing to profile the microbiota composition. The results show that cooking and digestion significantly affected the levels of PAs in all grains (*p* ≤ 0.05) compared to raw grains. Colonic fermentation resulted in a peak of total soluble PAs at 4–6 h for red sorghum and white corn and at 24 h for red millet and red fonio. *Enterobacteriaceae* genera were the most abundant at 24 h in all grains studied. 3-hydroxybenzaldehyde correlated positively with the relative abundance of *Dorea* and the mucus-degrader bacteria *Akkermansia* (*p* ≤ 0.05), whereas hydroferulic acid and isoferulic acid levels correlated negatively with *Oscillospira* and *Ruminococcus* (*p* ≤ 0.05), respectively. Our data indicate that cooking, digestion, and colonic fermentation affect the release of bound PAs from wholegrains and, consequently, their metabolic conversion. Furthermore, PA fermentation in the gut is associated with potentially relevant changes in the microbiota. This in vitro study provides the basis for the design of an in vivo human intervention study that can confirm the trends herein observed but also assess the impact on health outcomes.

## 1. Introduction

Cereal whole grains are part of the family *Poaceae* (also known as *Gramineae*), and their seeds are consumed as food. Wholegrains are a rich source of fibre and phenolic acids (PAs), and their consumption may be beneficial for the prevention of chronic illnesses, but this is subject to the bioaccessibility in the gastrointestinal tract and the subsequent bioavailability of their phenolic compounds [1]. In some continents, cereals serve as the major staple food for the population, and in Asia and Africa, sorghum and millet are known to be grown and consumed in addition to other grains [2]. Grains like fonio, sorghum, millet and corn are gluten-free and can be used as alternative grains in foods for different dietary needs. The nutritional value of the above grains is within the range of other grains such as wheat, oats and barley, with carbohydrate content that can vary from 63 to 86 g/100 g, proteins ranging from 6 to 12 g/100 g, and fats levels ranging from 0.5 to 5 g/100 g [3,4,5,6,7,8,9,10,11,12]. Fonio, millet, corn and sorghum are consumed widely in Nigeria and are cultivated in the Northern regions of the country. The grains are often consumed as fermented porridge, which is eaten by all age groups, from infants to adults and the elderly. They can also be prepared and consumed in different forms, including fermented drinks and boiled or roasted.

**Figure 1:**
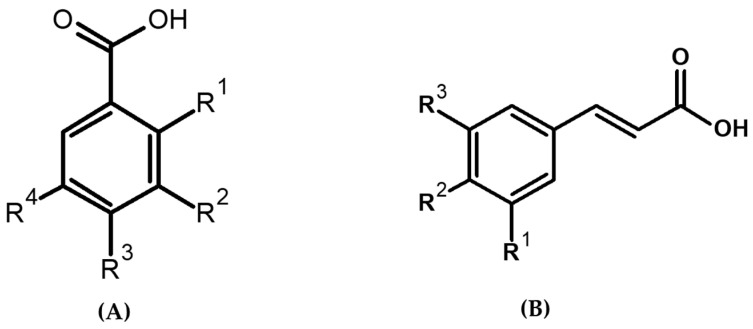
Basic structure of phenolic acids. (**A**) Hydroxybenzoic acid (vanillic acid (R2 = -OCH_3_, R3 = -OH), 4-hydroxybenzoic acid (R3 = -OH), salicylic acid (R1 = -OH), syringic acid (R3 = -OH, R2, R4 = -OCH_3_). (**B**) Hydroxycinnamic acid (p-coumaric acid (R2 = -OH), caffeic acid (R2, R3 = -OH), ferulic acid (R2 = OH, R3 = -OCH_3_), R1–R4 = H, except where specified.

PAs are a class of polyphenols consisting of an aromatic ring, a hydroxyl group and a carboxylic moiety. The two main groups of PAs are hydroxybenzoic acid (HBA) and hydroxycinnamic acid (HCA) (Figure 1). Phenolic compounds are known to possess bioactivity and health benefits [13].

There is no research information on the PA content and bioaccessibility of Nigerian grains; previous studies focus on the total phenolic compounds in the grains [14,15]. The aim of this study was to investigate the PA content of Nigerian-grown grains. A pilot study was performed, where a variety of grains, including red and white fonio, red and white millet, red and white sorghum, ofada rice and corn, were screened for their PA and total dietary fibre (TDF) content; based on these results, red fonio, red millet, red sorghum and white corn were selected for digestion and fermentation studies.

We investigated the bioaccessibility of selected PAs using a standardised in vitro digestion model [16]. Furthermore, following digestion, grains were subjected to a 24 h fermentation to evaluate the impact of their phenolic extract on human colonic microbiota and vice versa using an in vitro colonic model. Samples were obtained from the fermentation vessel at time points 0, 4, 6, 8 and 24 h to determine the PA profile of the aliquot, while DNA profiling was performed using NGS for samples obtained at time points 0, 6, and 24 h [17].

## 2. Results

### 2.1. Phenolic Acid and Total Dietary Fiber Content of Raw Grains

#### 2.1.1. UPLC-MS/MS Characterization of PAs

Supplementary Table S1 shows the MS detection parameters, and Supplementary Table S2 shows the calibration curve parameters for the 26 PA standards used in this study. For most compounds, at least three daughter fragments were generated from varying collision energies.

#### 2.1.2. Total PA and TDF Content of Raw Grains

The TDF of the grains ranged from 3 to 26.7 g/100 g (Table 1). Red sorghum had the highest, while white fonio had the lowest TDF values.

Grain PAs were extracted in soluble and bound forms, with total PAs calculated as the sum of soluble and bound PAs and expressed as ng/mg fresh weight. Table 1 shows the total PAs in raw grains. The highest value of total PA concentration was seen in white corn compared to other grains (2202.5 ± 34.9 ng/mg, *p* ≤ 0.0001). Apart from this, significantly high values of total PA concentration (*p* ≤ 0.0001) were also seen in red millet (1631.2 ± 227.5 ng/mg), white millet (1466.1 ± 187.1 ng/mg) and red sorghum (1464.2 ± 18.2 ng/mg) compared to the rest grains. White Sorghum had a total PA concentration of 693.3 ± 38.0 ng/mg, which was significantly higher (*p* ≤ 0.05) than that in Ofada rice and fonio, which had the lowest total PA concentrations, with values ranging from (132.2 ± 12.1 to 281.4 ± 10.8 ng/mg). PAs were mostly present in the bound form between 73.2% and 99.9% of total PAs. Millet and red sorghum had the highest proportions of soluble PAs (13.3% and 26.8%, respectively).

### 2.2. Impact of Cooking and Digestion on the Phenolic Acid Profile of Grains

Selected grains were cooked, subjected to simulated digestion and then freeze-dried. PAs were extracted from cooked and digested grains in soluble and bound forms, with total PAs calculated as the sum of soluble and bound PAs expressed as ng/mg fresh or dried weight. Table 2 shows the PA profile of raw, cooked, and digested grain samples.

#### Soluble and Bound PAs in Cooked and Digested Grains

**Figure 2:**
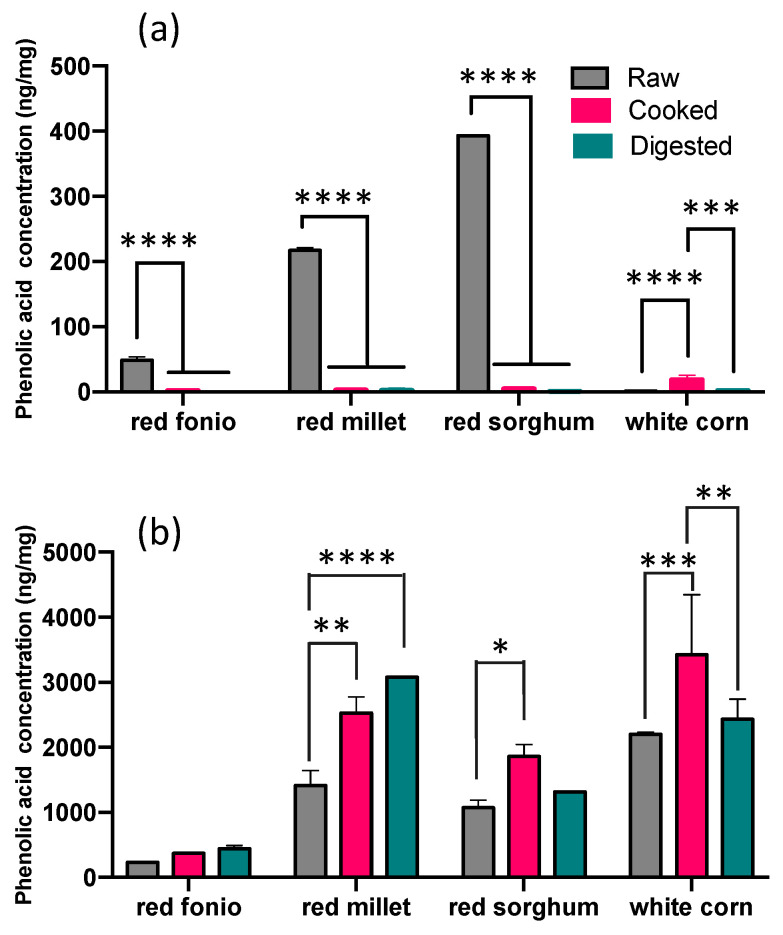
Total soluble (**a**) and bound (**b**) phenolic acids in raw, cooked, and digested grains. Raw samples were analysed in triplicate, cooked and digested samples in duplicate; all results are expressed as mean ± SD (2-way ANOVA, Tukey’s multiple comparisons tests, * = *p* ≤ 0.05, ** = *p* ≤ 0.01, *** = *p* ≤ 0.001, **** = *p* ≤ 0.0001).

The impact of cooking and digestion on the soluble and bound PAs of the grains can be seen in Figure 2. Cooking significantly reduced the total soluble amount of PAs in red fonio and also in red millet and red sorghum samples (*p* ≤ 0.05), whereas it led to a significant increase in white corn. The amount of soluble PAs measured in the grains after cooking varied from 1.8 ± 0.8 to 18.5 ± 7.1 ng/mg. The digested grains were also found to contain a lower amount of soluble forms of PA than raw grains, and this difference was significant for all grains (*p* ≤ 0.0001), with the exception of white corn. The levels of soluble PAs in digested grains ranged from 0.1 ± 0.03 to 2.3 ± 3.2 ng/mg.

The amount of bound PAs measured in cooked grains varied from 373.1 ± 8.0 to 3426.6 ± 920.1 ng/mg. The cooking process significantly increased the levels of bound PAs in all grains (*p* ≤ 0.05), with the exception of red fonio. The digestion process also had an impact on bound PA levels, and a significant increase was observed in red millet samples (*p* ≤ 0.001). We also noted how the digestion of white corn samples induced a significant decrease in the levels of bound PAs when compared to the cooked samples (*p* ≤ 0.01). Bound PA levels in digested grains varied from 444.5 ± 45.3 to 3075.4 ± 5.4 ng/mg.

**Figure 3:**
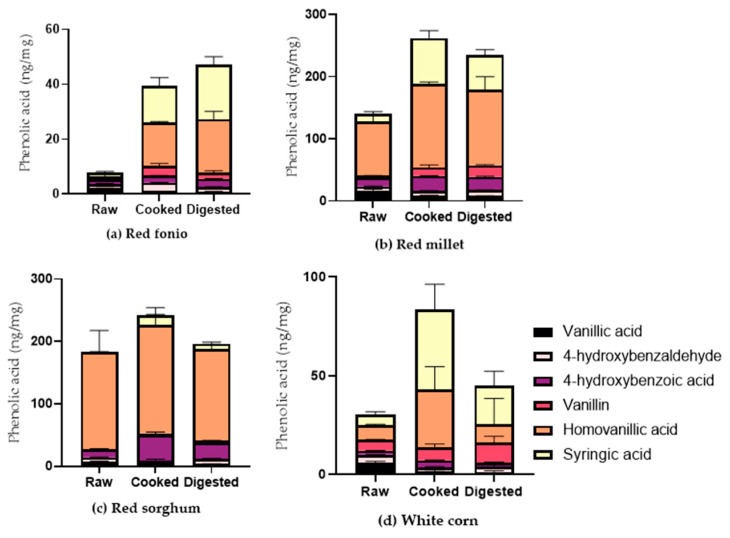
Amount of selected bound hydroxybenzoic acids in raw, cooked, and digested grains. Raw samples were analysed in triplicate, cooked and digested samples in duplicate. All results are expressed as mean ± SD.

The amount of selected bound HBAs in cooked and digested grains is shown in Figure 3. Homovanillic acid content was significantly higher in all grains after cooking, in comparison to the raw forms (*p* ≤ 0.01), whereas we can notice how the amount of other bound forms, such as HBAs, increased only in some cooked grains. For example, in white corn, we measured increased levels of syringic acid, which were also higher in red fonio samples (*p* ≤ 0.0001), whereas in red sorghum and millet, we observed increased levels of 4-OH benzoic acid (*p* ≤ 0.05). The levels of vanillin in red millet samples and fonio samples were also increased (*p* ≤ 0.05). In comparison to raw grains, the digestion process also affected the levels of PAs, with increases in syringic acid in white corn, red millet and red fonio (*p* ≤ 0.0001) and increases in vanillin and homovanillic acid in red millet and red fonio (*p* ≤ 0.05). The increase in homovanillic acid and syringic acid in red fonio was also significant when compared to cooked samples (*p* ≤ 0.0001). Conversely, the digestion process resulted in a significant decrease in homovanillic acid levels in white corn (*p* ≤ 0.0001), sorghum (*p* ≤ 0.0001) and red millet (*p* ≤ 0.05), and decreased levels of 4-OH benzoic acid in sorghum (*p* ≤ 0.05), as well as a decrease in syringic acid levels in red millet (*p* ≤ 0.0001), compared to cooked grains.

**Figure 4:**
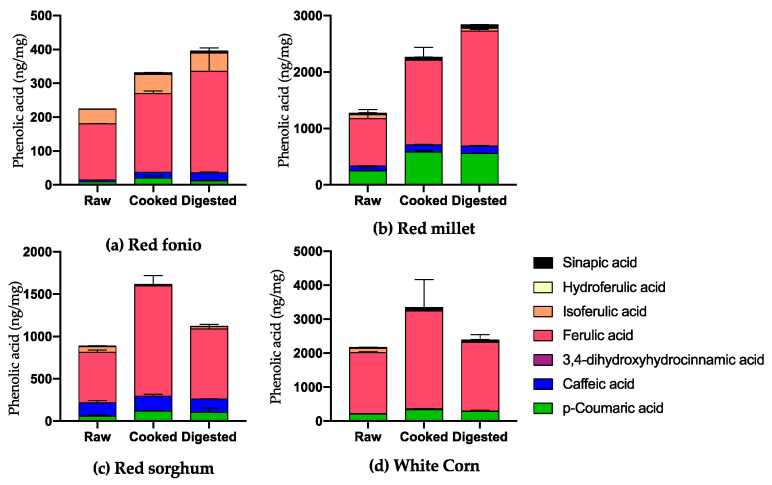
Amount of selected bound hydroxycinnamic acids in raw, cooked and digested grains. Raw samples were analysed in triplicate, cooked and digested samples in duplicate. All results are expressed as mean ± SD.

For selected bound HCAs (Figure 4), the cooking process significantly increased ferulic acid levels in all grains (*p* ≤ 0.0001). Additionally, a significant increase in p-coumaric acid levels was measured in red millet samples (*p* ≤ 0.001). In comparison to the raw samples, the red fonio, sorghum and red millet grains subjected to digestion contained significantly higher amounts of ferulic acid (*p* ≤ 0.0001), whereas the red millet samples contained significantly higher levels of p-coumaric acid (*p* ≤ 0.0001). In comparison to the cooked samples, the digested ones were found to contain a significantly higher amount of ferulic acid for some grains (red millet and red fonio) but a significantly lower amount for others, such as sorghum and white corn (*p* ≤ 0.001).

### 2.3. In Vitro Colonic Fermentation of Digested Grains

#### 2.3.1. Phenolic Acid Content of Fermented Grains

**Figure 5:**
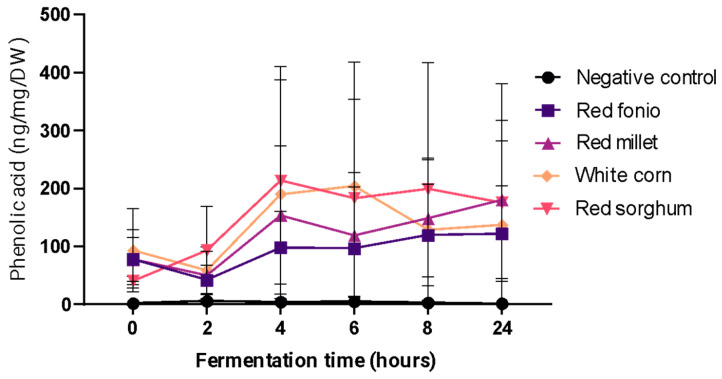
Total soluble phenolic acid concentration at different time points during in vitro colonic fermentation of digested grains. Samples were analysed in triplicate, except for red millet at 0 h, white corn at 6 h, and red sorghum at 0, 4, and 6 h, which were analysed in duplicate. All results are expressed as mean ± SD. DW, dry weight.

Digested grains were subjected to in vitro colonic fermentation for 24 h, and aliquots were collected at 0, 2, 4, 6, 8, and 24 h for assessment of soluble PA content (Figure 5). The total soluble PA content did not differ significantly over time for any of the grains (*p* > 0.05), but the highest levels were measured at different time points for different grains (4 h, 6 h or 24 h for red sorghum, white corn, or red fonio and red millet, respectively. The total soluble PA forms released from each grain were significantly different in white corn, red sorghum and red millet in comparison to the negative control group (*p* ≤ 0.05).

**Figure 6:**
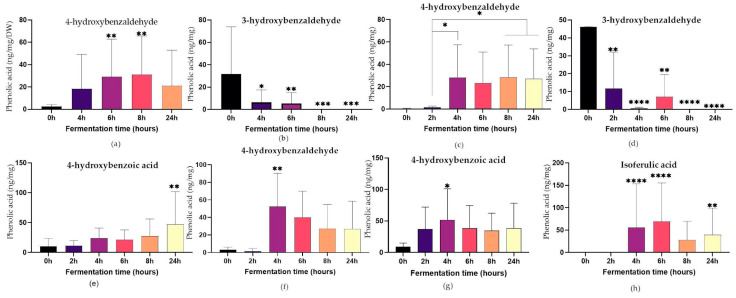
Changes in selected phenolic acids in red fonio (**a**,**b**), red millet (**c**–**e**), red sorghum (**f**,**g**) and white corn (**h**) during in vitro colonic fermentation of digested grains. 2-way ANOVA, Tukey’s multiple comparisons tests, each time point vs. 0 h (unless otherwise indicated), * = *p* ≤ 0.05, ** = *p* ≤ 0.01, *** = *p* ≤ 0.001, **** = *p* ≤ 0.0001.

Looking at the changes in selected PAs over the fermentation period, 4-OH benzaldehyde increased significantly in red fonio, red millet, and red sorghum (*p* ≤ 0.05) (Figure 6). In particular, peak levels of 4-OH benzaldehyde were observed at 6 h in red fonio and red sorghum and at 24 h in red millet. 4-OH benzoic acid levels were significantly higher in red millet and red sorghum (*p* ≤ 0.05), with peak values measured after 24 h in both grains. In addition, the fermentation of white corn substrate resulted in the production of Isoferulic acid, which was significant (*p* ≤ 0.05) after 4 h, 6 h, and 24 h.

#### 2.3.2. Impact of Digested Grains on Faecal-Derived Microbial Communities from Healthy Volunteers

**Figure 7:**
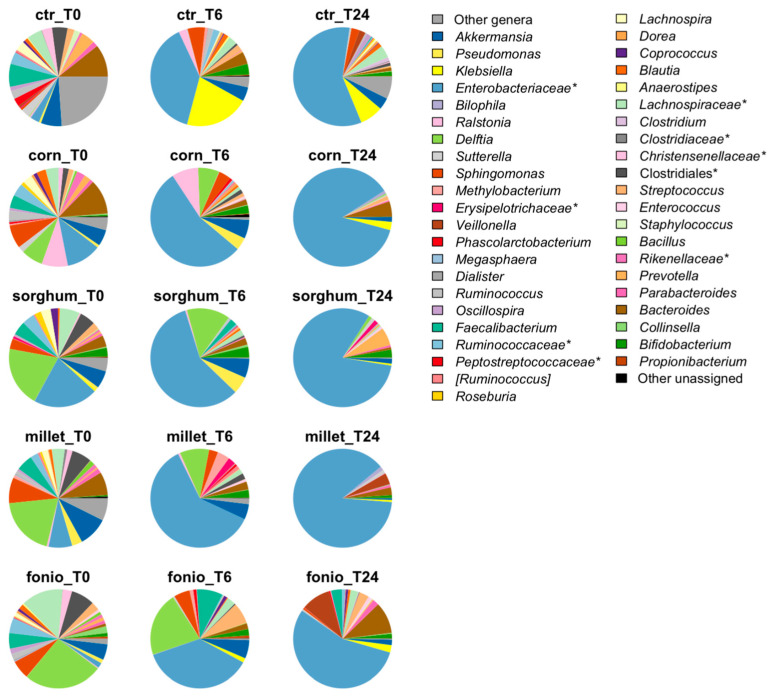
Genus-level relative abundance profiles of faecal microbial communities from healthy volunteers before (T0) and after 6 (T6) and 24 (T24) h of in vitro colonic fermentation with digested grains. Ctr, negative control (i.e., faecal slurry without any substrate addition). * = bacterial family with unclassified genera.

The faecal microbial communities of three healthy volunteers were profiled over time during in vitro colonic fermentation of digested grains to assess whether the different dynamics of PA conversion were associated with different gut microbiota layouts and trajectories (Figure 7). The diversity of the microbial communities decreased remarkably over time for all grains, in parallel with an increase in the relative abundance of unclassified *Enterobacteriaceae* (relative abundance, 37% to 61% at 6 h, 55% to 87% at 24 h), particularly for white corn and red millet. The highest diversity at 24 h was found for red fonio, whose microbial communities were mainly composed of unclassified *Enterobacteriaceae* (55%), *Bacteroides* (10%), and *Veillonella* (9%).

**Figure 8:**
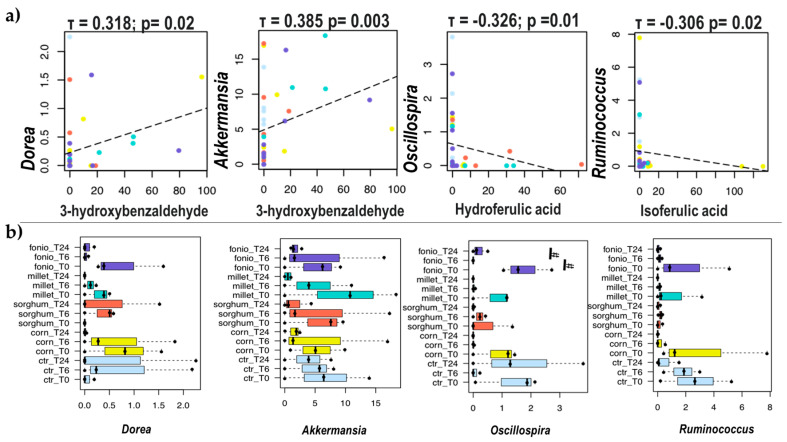
Associations between bacterial genera and phenolic acids during in vitro colonic fermentation of digested grains. (**a**) scatter plots showing the correlation between genus-level relative abundances and levels of selected phenolic acids. Only statistically significant correlations (*p* ≤ 0.05) with absolute Kendall’s tau ≥ 0.3 are shown. Samples are coloured based on the digested grains subjected to fermentation (see bottom panel). (**b**) Boxplots showing the relative abundance distribution of the genera significantly correlated with PAs. For each digested grain, samples were collected before (T0) and after 6 (T6) and 24 (T24) h of fermentation. Ctr, negative control (i.e., faecal slurry without any substrate addition). Wilcoxon test, # = *p* ≤ 0.1.

Correlation analysis between selected PAs and bacterial genera revealed some trends (Figure 8). In particular, 3-hydroxybenzaldehyde was positively correlated with *Dorea* and the mucus-degrader bacteria *Akkermansia* (*p* ≤ 0.05), whereas both hydroferulic and isoferulic acids showed a negative correlation with the bacteria *Oscillospira* and *Ruminococcus*, respectively (*p* ≤ 0.05). Both *Dorea* and *Oscillospira* tended to decrease over time in red fonio (*p* ≤ 0.1), while *Ruminococcus* tended to decrease in white corn (*p* ≤ 0.1).

## 3. Discussion

Dietary PAs have been found to beneficially affect health, but their bioaccessibility can have a significant impact on bioavailability and absorption. In this study, we provided a comprehensive and accurate assessment of extractable PAs in both soluble and bound forms in a panel of selected grains. The assessment was provided in raw grains but also in processed forms of nutritional interest (following cooking and simulated digestion), applying robust and validated analytical methods. As dietary fibre can bind and influence the mobility, transition and absorption of PAs in the digestive tract [18,19], ultimately affecting their bioavailability, the TDF content of the grains was assessed.

The TDF content of grains in this study was found to be as follows: red and white sorghum > white corn > red and white millet > ofada rice > red and white fonio. Our results are generally consistent with the available literature, which reports values of 3.3–8% in hulled fonio, 3.7–15% in pearl millet, 8.6–21.0% in sorghum, 1.4–3.5% in ofada rice, 11.1–15.34% and 2.1–10.1% in corn [3,4,5,6,7,8,9,10,11,12,20,21,22]

The total PA content of the grains selected for analysis in this study ranged from 276.64 to 2202.5 ng/mg, as the total PA amount in wholegrains can differ subject to the grain type, the environment and farming methods, as well as the different types of processing applied, the PAs selected for characterisation, as well as the extraction procedures and analytical methods applied. One study showed that total PAs differed significantly based on the type of corn [23] and rice cultivars [24]. Other studies showed differences in total PAs by grain type; for example, total PA content was 61.82 µg/g in raw fonio, 871.48 µg/g in raw millet, 678.36 µg/g in raw sorghum, and 832.2 µg/g in raw pearl millet [25,26].

The impact of cooking on the PA content in grain samples can vary in relation to several factors, including processing, for example, milling, and cooking procedures, including cooking duration and temperature levels, which could affect the bioaccessibility and amount of extractable PAs. During the process of cooking, the soluble PAs could be leached into the cooking solution, making them susceptible to thermal degradation, or they could form complexes within the food matrix, causing a decrease in their amount. Furthermore, during the cooking process, cell wall polymers may, in turn, be broken down to facilitate the extraction of bound PAs [26,27]. Indeed, our results showed that cooking led to a decrease in soluble PAs and an increase in bound PAs in most grains. However, there is conflicting data in the literature. For example, a previous study found that cooking increased soluble PAs in sorghum and millet [26]. Differences in methods, especially cooking time (12 min for sorghum and 15 min for millet compared to 43 min and 36 min in this study), may explain the different observations. The study also reported that cooking fonio caused an increase in bound PAs, while cooking sorghum caused a decrease in bound PAs, with syringic and salicylic acids detected only in the bound cooked fractions [26]. A different study [28] observed that cooking durum wheat caused an increase in its bound PA content, and the authors suggested that this increase correlated with the increased antioxidant activity of their cooked samples. Other studies record that cooking waxy corn and rice caused a decrease in PA content in these grains [27,29]. Cooking was also reported to increase the total amount of free PAs [24].

The data presented in our study indicate how in vitro gastrointestinal digestion can reduce total soluble and increase total bound PA compared to raw grains. Literature data also indicate how boiling together with digestion can have an impact on PA levels, increasing the levels of ferulic acid in the intestinal digest compared to the digest of unprocessed sorghum samples [30]. The degradation of soluble PAs in the gastrointestinal tract (affected by the pH and also by the interaction with digestive enzymes) could result in the formation of new metabolic entities and a reduction in detectable soluble PA. Bond formation with cell wall constituents and resultant conversion or confinement of soluble PA may be a factor in the reduction in soluble PAs in digested samples. On the other hand, bound PAs can be more durable in the upper gastrointestinal tract due to their stronger association with cell wall structures, and the loosening of these structures during cooking, digestion or extraction may facilitate increased accessibility for extraction of bound PAs [26,30,31,32].

Bound PAs are not readily accessible in the upper part of the gastrointestinal tract, but in the lower part, the microbiota may also play a role, affecting their bioaccessibility and facilitating their release from the food matrix and subsequent biotransformation. Therefore, as part of the current study, we simulated the colonic fermentation of digested food in a controlled in vitro environment, which provided valuable insights into the bioaccessibility of PAs as well as other compounds. In this regard, several studies in the literature report changes in PA levels during in vitro human colonic fermentation of foods high in PA. For example, increases in protocatechuic acid and syringic acid and a decrease in vanillic acid levels have been observed, in addition to a gradual release of PAs from food substances supplemented with PA, while other reports show a decrease in PAs during the fermentation period [33,34,35,36,37]. Inter-individual variability in participants’ gut microbiota is well known, and in combination with differences in the food matrix or experimental methods, may be an explanation for these differences in results.

In this study, after the cooking and digestion processes, the colonic fermentation process was also applied to the digested grain material using a validated in vitro intestinal model. Our results indicate that the total soluble PA content varies during the fermentation process, with a peak at 4–24 h; during this period, there was a gradual release of PAs measured during the 24 h fermentation period. Additionally, the analysis of pre-fermented samples also indicated a high content of bound PA (which was considered not bioaccessible prior to fermentation). The impact of the interaction between the bound PA forms and the food matrix and the resulting release of soluble forms from the matrix observed during the fermentation process could be explained as a result of the extended release of soluble PAs in the colonic environment. In addition, some specific PAs significantly decreased or increased over time during fermentation, while others were not detected in the fermented samples. For example, at 0 h, 3-hydroxybenzaldehyde was the highest PA observed in all the grains studied. Thus, over time, its levels decreased significantly in red fonio samples, as well as in red millet samples, during the 24-h fermentation period. It can be speculated that it was utilised or metabolised to produce a different end product. On the other hand, 4-OH benzaldehyde, 4-OH benzoic acid, and isoferulic acid levels were observed to increase significantly during the 24-h fermentation period in some grains. This might suggest their release from the fibre into soluble forms or their synthesis through the biotransformation of other phenolic compounds in the grains by the action of the gut microbiota.

Once in the large intestine, the bound PA forms are degraded under the action of esterase activities, and the final metabolic product is likely to result from the synergistic action of the gut microbial taxa present. Biotransformation reactions that are likely to occur in the large intestine and affect the phenolic compounds present in the digesta can include hydrolysis, demethylation, reduction, decarboxylation, dihydroxylation and β-oxidation. These reactions can be carried out using bacterial species from the genera *Bifidobacterium* (phylum Actinobacteria), *Eggerthella*, *Lactobacillus*, *Clostridium*, *Eubacterium*, *Streptococcus*, *Ruminococcus* (phylum Firmicutes) *Peptostreptococcus,* and *Escherichia* (phylum Proteobacteria) [38,39,40,41]. β-oxidation reactions can also occur and cause the reduction in the hydrocarbon side chains in HCAs. A combination of different microbiota species, such as *Escherichia coli* and *Enterococcus faecalis*, was found to be responsible for the caffeic acid conversion to 3-hydroxyphenylpropionic acid, while benzoic acids can be measured as the end product [42]. A plausible pathway for the biotransformation of HCAs through the action of gut microbes is the degradation of HCAs to phenylpropionic acid derivatives, where demethylation can also occur. The formed metabolic derivatives can then be subjected to a series of β-oxidations to produce benzoic acids [43].

The results of in vitro colonic fermentation experiments showed an overall predominance of unclassified *Enterobacteriaceae* members at 6–24 h in all study groups, as reported in oat fermentation studies [44]. *Enterobacteriaceae* species are able to metabolise ferulic acid to produce vanillin [45]. In our study, prior to fermentation, soluble ferulic acid levels were seen in low amounts in all the forms of the grains, but these grains contained high levels of bound ferulic acid. For most grains, relatively low levels of soluble ferulic acid were also measured, except for white corn (14.57 ± 16.49 ng/mg at 6 h). It is, therefore, possible to hypothesise that, to some extent, the bound forms of ferulic acid may have been biotransformed into other products. For example, after fermentation of the white corn substrate, high levels of isoferulic acid were measured, whereas after fermentation of red millet and red sorghum substrates, high levels of hydroferulic acids were measured, probably suggesting that some of the bound ferulic acids were biotransformed to these related compounds. The microbiota analysis also showed reduced proportions of *Ruminococcus* spp. at 6 h in white corn. Interestingly, the relative abundance of *Ruminococcus* spp. correlated negatively with isoferulic acid. As anticipated, white corn had the highest amount of isoferulic acid compared to other grains during the fermentation period, with a peak at 6 h (69.55 ± 85.68 ng/mg), suggesting a possible inhibitory effect against *Ruminococcus*. No data are available in the literature, but it should be noted that ferulic acid, which was the highest in white corn at 6 h as well, can suppress the growth of cellulolytic bacteria, including *Ruminococcus* spp. [46].

A prolonged positive effect of the soluble PA compounds in the colonic environment may emerge from the progressive release of soluble PA from the food matrix throughout the fermentation process. Additionally, the ability of the microbiota to alter PAs results in a variety of metabolites that, if taken into systemic circulation, may be advantageous for colonic health and may produce advantageous bioactivity in vivo. The health benefit of phenolic compounds, including PAs, may be attributed to their antioxidant and anti-inflammatory nature, which makes them relevant in the prevention of chronic illnesses [47].

In addition to antioxidant and anti-inflammatory benefits, phenolic compounds exhibit antibacterial, neuroprotective, anticancer, and cardioprotective functions [48]. For instance, ferulic acid’s metabolic product vanillic acid has been shown to have antibacterial, anticancer, and anti-inflammatory characteristics, while ferulic acid-4-O-sulfate and dihydroferulic acid have also been shown to have anti-inflammatory and cardioprotective qualities [49,50,51,52,53]. According to a study, pro-inflammatory cytokines were observed to be inhibited using derived phenolic acid metabolites [41].

Research is still ongoing to determine the precise mechanisms underlying these actions, but some indications suggest that phenolic compounds and their metabolites may act as antioxidants by linking with free radicals and extinguishing them while promoting the production of endogenous antioxidant enzymes [48]. They also function as antimicrobial agents by creating hydrogen bonds with bacterial cell membrane bilayers or interacting with cell wall constituents to compromise the microbial cells’ barrier of defence. Additionally, they might bind toxins and disrupt the bacterial cell’s energy metabolism [54,55].

Finally, in this study, 3-hydroxybenzaldehyde, which decreased over time in red fonio and red millet, had a positive correlation with *Akkermansia*. This bacterial genus has been shown to promote metabolic health (and is now available as a postbiotic for weight management and glycemic control), but its ability to degrade mucus raises questions about its overall safety [56,57].

## 4. Materials and Methods

### 4.1. Chemical Reagents

Twenty-six PA compounds and some potential metabolites; (vanillic acid (VA), isovanillic acid (IVA), 4-hydroxybenzaldehyde (4-HBALD), 3-hydroxybenzaldehyde (3-HBALD), 4-hydroxybenzoic acid (4-HBA), 3-hydroxybenzoic acid (3-HBA), salicylic acid (SA), 4-hydroxyphenylacetic acid (4-HPA), 3-hydroxyphenylacetic acid (3-HPA), 2-hydroxyphenylacetic acid (2-HPA), vanillin (VN), 3,4- and 3,5-dihydroxybenzoic acid sum (3,4- and 3,5-DHBAs), 2,5-dihydroxy benzoic acid (2,5-DHBA), 2,4-dihydroxybenzoic acid (2,4-DHBA), p-coumaric acid (pCA), gallic acid (GA), hippuric acid (HA), caffeic acid (CA), 3,4-dihydroxyhydrocinnamic acid (3,4-DHCA), homovanillic acid (HVA), syringaldehyde (SYRALD), ferulic acid (FA), isoferulic acid (IFA), hydroferulic acid (HFA), syringic acid (SYRA), and sinapic acid (SNA), 3,5-dichloro-4-hydroxybenzoic acid (used as internal standard), bile extract, pancreatin, α-amylase and pepsin were purchased from Sigma-Aldrich (Gillingham, UK). Analytical grade NaOH, CaCl_2_, and HCl were also used, in addition to LC-MS grade solvents (formic acid, ethyl acetate, acetonitrile methanol, ethanol) and water. Peptone water (BDH, Poole, UK), yeast extract (Oxoid, Basingstoke, UK), NaCl, K_2_HPO_4_ (BDH), KH_2_PO_4_ (BDH), MgSO_4_ × 7H_2_O (Fisher Scientific, Loughborough, UK), CaCl_2_ × 6H_2_O (0.01 g/L), NaHCO_3_ (Fisher Scientific), tween-80, hemin (0.05 g/L), vitamin K (10 µL/L), L-cysteine HCl (0.5 g/L), bile salts (0.5 g/L) (Oxoid, Basingstoke, UK), resazurin solution 0.025 g/100 mL (4 mL/L, pH 7), QIAamp DNA Stool Kit (QIAGEN, Hilden, Germany), phosphate-buffered saline (Oxoid, Basingstoke, UK), inulin (P95-FOS, Orafti, Tienen, Belgium), were also used.

### 4.2. Wholegrains

All grains analysed in this study were purchased from a Nigerian market located in Jos (9.8965° N, 8.8583° E). The grains included red and white varieties of fonio (*Digitaria* spp. *Iburua* and *exilis*) (also known as acha), red and white varieties of millet (*Pennisetum glaucum*), red and white varieties of sorghum (*Sorghum bicolor*), white corn (*Zea mays*) (also known as maise), and ofada rice (*Oryza glabberimma*).

### 4.3. Total Dietary Fibre (TDF) Determination

The total dietary fibre (TDF) levels of the wholegrain samples were determined using the Association of Official Analytical Chemists (AOAC) Official method 991.43 [58]. The results were expressed as g/100 g.

### 4.4. Wholegrain Processing (Cooking and Digestion)

Raw grains were milled and used for PA extraction. A preliminary screening analysis of the raw grains was made and led to the selection of the 4 grains utilised for the study. The selection was made to include grains with varied fibre and PA contents. Selected grains (red fonio, red millet, red sorghum and white corn) were further studied to assess the impact of cooking and in vitro digestion on PA levels. Selected raw samples (100 g) were weighed and boiled in water until tender, with cooking times of 15 min, 36 min, 43 min and 79 min for red fonio, red millet, red sorghum and white corn, respectively (Table 3). After cooking, the samples were kept at −20 °C until digestion.

After cooking, the samples were further processed in order to simulate the different phases of the digestion process (oral, gastric, and intestinal), with a procedure similar to the one described in [16] with minor adjustments. The oral phase of the digestion was performed as follows: a 10 g aliquot of milled and boiled samples was mixed with distilled water, and α-amylase was added (5 mg dissolved in 10 mL of 1 mM CaCl_2_). The mixture was then kept at 37 °C for 30 min on a shaker. The gastric digestion phase was then simulated by adjusting the pH of the oral digest to 2 and adding 12 M HCl. Pepsin (51.2 mg/mL of sample) was then added, and the mixture was incubated at 37 °C for 2 h on a shaker. After incubation, the pH was increased to 7.5 to simulate the small intestinal conditions, using 6 M NaOH. Pancreatin (4 mg/mL of sample) and bile extract (25 mg/mL of sample) were added, and the mixture was kept at 37 °C for 2 h on a shaker. The final digested material of each sample was subsequently freeze-dried and kept at −20 °C until the subsequent analysis for PA characterisation by UPLC-MS/MS and in vitro colonic fermentation.

### 4.5. Colonic Fermentation

#### 4.5.1. Basal Media Composition

The basal media was made by mixing yeast extract (Oxoid, Hampshire, UK), Peptone water (BDH, Poole, UK), KH_2_PO_4_ (BDH), MgSO_4_ × 7H_2_O NaCl, K_2_HPO_4_ (BDH), (Fisher Scientific, Loughborough, UK), CaCl_2_ × 6H_2_O (0.01 g/L), NaHCO_3_ (Fisher Scientific), tween-80, hemin (0.05 g/L), vitamin K (10 µL/L), L-cysteine HCl (0.5 g/L), bile salts (0.5 g/L) (Oxoid, Basingstoke, UK) and resazurin solution 0.025 g/100 mL (4 mL/L, pH 7). The obtained solution was heated, cooled, poured into Duran bottles, autoclaved and stored until use.

#### 4.5.2. In Vitro Fermentation

Faecal samples obtained from three healthy volunteers who were not taking any medications or supplements were used for this study. Faecal samples were diluted (1:10 *w*/*v*) in phosphate-buffered saline (0.1 mol/L phosphate-buffered solution, pH 7.4) and homogenised (Stomacher 400, Seward, UK) for 2 min at 240 paddle beats per min in vitro colonic fermentation was performed according to [17] in an anaerobic pH-controlled batch culture system. Five vessels were used for each faecal slurry; four of these vessels were used to ferment the digested grains (red fonio, red millet, red sorghum and white corn), and one vessel for the negative control, i.e., faecal slurry without any substrate addition. Each vessel was inoculated with 5 mL of fresh faecal slurry (1/10 *w*/*w*). Digested freeze-dried grain substrates (1% *w*/*w* dry solid/total dietary fibre) prepared in basal medium (full description of the basal medium composition is indicated in 2.5.1) were fermented. Batch cultures were run in three replicates (n = 3) over a period of 24 h, and samples were collected from each vessel at 0, 2, 4, 6, 8 and 24 h for PAs extraction. Microbiota profiling was performed for 0, 6 and 24 h aliquots.

### 4.6. PAs Extraction and Analysis

PAs were extracted in soluble (free and conjugated) and bound fractions from 100 mg of raw and digested milled grains according to the method of [59] with slight modifications. An aliquot of 100 mg of the milled whole grain samples was used for the extraction procedure, and it was spiked with 5 µL of internal standard solution. 1 mL of 80% water/ethanol solution was also added to the solution. The solution was agitated with a vortex mixer and then sonicated for 10 min. The obtained solution was then centrifuged at 13,200 rpm for 15 min. The supernatant was collected, 1 mL of 80% ethanol was added, and the process was repeated to extract the phenolic acids. The residue was collected and used for the extraction of bound phenolic acids, while the combined supernatants were evaporated with nitrogen steam. The samples were then further processed in order to extract the conjugated phenolic acids as follows: a 400 µL aliquot of 2 M NaOH was added, and the samples were agitated and kept in the dark for 4 h. After this step, the solution was acidified by adding 80 µL 12 M HCl and then vortexed. 500 µL of ethyl acetate was added, and the sample was mixed again to facilitate extraction of the PAs. The solution was centrifuged (13,200 rpm, 5 min), and the supernatant was collected and evaporated. Ethyl acetate (500 µL) was added to the samples, and the extraction process was repeated again. The supernatant was then collected and combined with the first supernatant aliquot and dried under a nitrogen stream. The dried samples were kept at −20 °C until the subsequent PAs analysis. Before the extraction of bound PAs, 10 µL of internal standard was added to the residue. An alkaline hydrolysis procedure was performed with the addition of 400 µL of NaOH (2 M). The samples were kept in the dark for 4 h during the hydrolysis. Following this step, an aliquot of HCl (120 µL) was added to the solution to adjust the pH. The PAs were then extracted in ethyl acetate (800 µL) and centrifuged (13,200 rpm, 5 min) twice. The two supernatants were combined and fully evaporated under a nitrogen stream. The evaporated samples were kept at −20 °C until the time of analysis.

The process described above was used for the extraction of PAs from cooked samples; 100 mg equivalent of cooked grains was used for the extraction. Fermented samples (200 µL) were extracted and assayed for soluble PAs as described above; extracts were evaporated to dryness and purified using solid phase extraction (see below).

#### 4.6.1. Solid Phase Extraction (SPE)

The evaporated samples were reconstituted by adding 0.5 mL acidified water (1% formic acid) and subjected to SPE as follows: Phenomenex SPE cartridges (StrataTM-X 33 µm, polymeric, reverse phase, 100 mg 3 mL^−1^) were used, and preconditioned by adding 3 mL acidified methanol (1% formic acid), followed by 3 mL acidified water. The acidified water was allowed to elute through the cartridge, and 0.5 mL of acidified water was then added to the column. A 0.5 mL aliquot of reconstituted sample was also loaded onto the column and eluted through the column. The column was rinsed twice with 0.5 mL of acidified water and then washed twice with 6 mL of water before applying the vacuum. After the washing steps, the sample eluent was collected into clean tubes using acidified methanol (2.5 + 0.75 mL) as an eluting solvent, and the vacuum was then applied. The eluted samples were evaporated and reconstituted with acidified water (100 µL), vortexed (1 min), sonicated (2 min), mixed again (1 min), and transferred into a 96-well plate for UPLC-MS/MS analysis.

#### 4.6.2. Ultraperformance Liquid Chromatography-Tandem Mass Spectrometry (UPLC-MS/MS)

Samples were filtered using 0.45 µm Polytetrafluoroethylene (PTFE) membrane filters prior to injection into the UPLC system. The PAs characterised by the samples were performed on a Waters Acquity H class UPLC system coupled to a Waters Xevo TQ micro Mass spectrometer (Waters, Milford, MA, USA). The chromatographic separations were performed on sample aliquots (2 μL) injected on an Acquity UPLC HSS T3 1.8 μm column (2.1 × 100 mm) with an HSS T3 1.8 μm Vanguard pre-column at 45 °C (0.65 mL min^−1^ flow rate). The mobile phase consisted of 0.1% *v*/*v* formic acid/water (phase A) and 0.1% *v*/*v* formic acid/acetonitrile. (Phase B) and the gradient was 99% A, 99% A, 70% A, 5% A, 5% A, 99% A, and 99% A scheduled at 0, 1, 10, 12, 13, 13.10 and 16 min, respectively. Twenty-six PA standards were infused for MS tuning and Multiple Reaction Monitoring (MRM) method optimisation. Three fragments were targeted for each analyte, and the one with the most intense signal was used for quantification. Calibration curves were used for the quantification of each compound using analytical grade standards (0.05–100 µg/mL range) and plotted against peak areas; R^2^ values (all higher than 0.95), limit of detection (LOD) and limit of Quantification (LOQ) concentrations were estimated as part of the method development and validation, and signal to noise ratios limits were set to (S/N) ≥ 3 (LOD) and S/N ≥ 10 (LOQ). LC-MS data were analysed using MassLynx software (V4.1, Waters, Milford, MA, USA). Total PA content was reported as the sum of both soluble and bound forms of PAs and expressed as ng/g fresh or dry weight.

### 4.7. Gut Microbiota Profiling

The gut microbiota profiling of the fermented samples was performed using the QIAamp DNA Stool Kit (QIAGEN, Hilden, Germany) on the DNA extracted from sample aliquots (250 mg) according to the kit instructions. The DNA concentration was measured on a NanoDrop ND-1000 spectrophotometer (NanoDrop Technologies, Thermo Scientific, Waltham, MA, USA). 16S rRNA amplicon sequencing and bioinformatics were applied as previously reported [17].

### 4.8. Statistical Analysis

PA analyses were performed in datasets for analysis conducted in triplicate for each raw and fermented sample and in duplicate for cooked and digested samples. The statistical analysis for all PA data was conducted on GraphPad Prism version 8 software (GraphPad Software, Boston, MA, USA). One-way ANOVA was performed to determine any significant differences in PA levels between grains, whereas a two-way ANOVA was performed to verify any significant effect of cooking and digestion on PA levels. Post hoc Tukey’s multiple comparison test (*p* ≤ 0.05) was used for both analyses. All results have been reported as mean ± SD.

Statistical analysis for the gut microbiota was performed as reported by [17], using the packages stats and vegan on R software (v4.2.0, R Foundation for Statistical Computing, Vienna, Austria). Non-parametric tests (Kruskal–Wallis test followed by post hoc Wilcoxon test) were performed using the stats package. Kendall rank correlation test was used to assess the associations between genus-level relative abundances and PA levels. Only statistically significant correlations with absolute Kendall’s tau ≥ 0.3 were considered. A *p*-value ≤ 0.05 was considered statistically significant; a *p*-value between 0.05 and 0.1 was considered a trend.

## 5. Conclusions

In this study, we investigated the PA profile of specific Nigerian-grown grains and further investigated how cooking, in vitro gastrointestinal digestion and in vitro colonic fermentation affected the PA profiles in selected grains. Our results indicated that in vitro gastrointestinal digestion of grains induced a decrease in total soluble PAs and an increase in total bound PA when compared to raw grains. The appearance of new metabolic forms not measured in the raw grains was also observed. Our data also suggest a relationship between the PA profiles of Nigerian grains and the configuration of the gut microbiota. However, further studies are needed to investigate the directionality of this relationship and the underlying mechanisms. Our data indicate how the PAs in the grains were able to significantly modulate the large intestine environment, and the current findings provide a robust starting point for further confirmation of the observed trends. Our findings provide a basis for a future in vivo study that also focuses on the potential beneficial effects of grains and their PAs on host health.

## Figures and Tables

**Table 1:** Total phenolic acid content, percentage of total soluble and bound phenolic acid fractions, and total dietary fibre in raw grains.

Raw Grain	Total PAs (ng/mg) FW	% Soluble PAs	% Bound PAs	TDF (g/100 g)
Red fonio	281.4 ± 10.8 ^a^	17.2 ± 1.9	82.8 ± 1.9	6.7
White fonio	132.2 ± 12.1 ^a^	5.1 ± 0.6	94.9 ± 9.7	3
Red millet	1631.2 ± 227.5 ^b^	13.3 ± 0.2	86.7 ± 14.1	11.4
White millet	1466.1 ± 187.1 ^b^	13.6 ± 1.0	86.4 ± 12.0	9.7
Red sorghum	1464.2 ± 184.2 ^b^	26.8 ± 0.0	73.2 ± 8.0	26.7
White sorghum	693.3 ± 38.0 ^c^	0.1 ± 0.0	99.9 ± 5.5	21.7
White corn	2202.5 ± 34.9 ^d^	0.1 ± 0.0	99.9 ± 1.6	14.3
Ofada rice	276.64 ± 25.31 ^a^	0.7 ± 0.3	99.3 ± 9.2	2.7

Samples were analysed in triplicates; results are expressed as mean ± SD. FW = fresh weight; PAs = phenolic acid; TDF = total dietary fibre. Values with different superscripts (a, b, c, d) differ significantly (*p* ≤ 0.05).

**Table 2:** Individual phenolic acids (soluble + bound) in raw, cooked, and digested grains (ng/mg fresh or dried weight).

		Red Fonio			Red Millet			Red Sorghum			White Corn	
PA	Raw	Cooked	Digested	Raw	Cooked	Digested	Raw	Cooked	Digested	Raw	Cooked	Digested
VA	6.82 ± 1.33	1.02 ± 0.05	0.83 ± 0.12	37.60 ± 1.92	7.94 ± 1.23	8.20 ± 0.33	33.00 ± 3.52	4.60 ± 0.27	3.49 ± 0.45	5.87 ± 0.73	1.85 ± 0.66	1.28 ± 0.56
IVA	ND	0.00 ± 0.00	ND	ND	ND	ND	ND	ND	ND	ND	ND	ND
4-HBALD	2.25 ± 0.14	3.47 ± 0.53	1.69 ± 0.10	7.63 ± 0.84	8.10 ± 1.13	9.19 ± 0.42	15.54 ± 0.31	7.52 ± 2.67	9.36 ± 0.70	4.37 ± 0.08	3.20 ± 0.18	3.01 ± 0.72
3-HBALD	ND	ND	ND	ND	ND	ND	ND	ND	ND	ND	ND	ND
4-HBA	4.76 ± 0.41	2.57 ± 0.02	3.07 ± 0.03	43.58 ± 4.44	25.13 ± 0.63	21.22 ± 1.20	39.99 ± 0.02	41.57 ± 5.24	26.34 ± 2.10	2.03 ± 0.07	3.80 ± 0.24	1.92 ± 0.33
3-HBALD	0.05 ± 0.07	ND	0.03 ± 0.04	ND	ND	ND	ND	ND	ND	ND	ND	ND
SA	0.46 ± 0.01	0.55 ± 0.04	0.24 ± 0.04	0.29 ± 0.05	0.30 ± 0.09	0.27 ± 0.05	0.43 ± 0.01	0.30 ± 0.06	0.13 ± 0.00	0.26 ± 0.06	1.14 ± 0.04	0.40 ± 0.00
4-HPA	ND	ND	ND	ND	ND	ND	ND	ND	ND	0.26 ± 0.37	ND	ND
3-HPA	ND	ND	ND	ND	ND	ND	ND	ND	ND	ND	ND	ND
2-HPA	0.03 ± 0.04	ND	ND	0.41 ± 0.02	ND	ND	ND	ND	ND	ND	ND	ND
VN	0.50 ± 0.18	3.78 ± 1.15	2.32 ± 0.73	2.91 ± 0.20	13.92 ± 4.17	18.56 ± 1.49	1.08 ± 0.26	2.87 ± 1.82	2.94 ± 0.12	5.53 ± 0.21	9.57 ± 0.85	10.93 ± 3.58
3,4- and 3,5-DHBAs	0.37 ± 0.04	0.44 ± 0.04	0.46 ± 0.02	2.04 ± 0.03	1.11 ± 0.08	1.33 ± 0.14	2.46 ± 0.02	1.85 ± 0.01	1.65 ± 0.23	0.06 ± 0.02	0.18 ± 0.01	0.10 ± 0.04
2,5-DHBA	ND	ND	0.03 ± 0.05	0.03 ± 0.03	0.01 ± 0.02	ND	ND	0.05 ± 0.05	0.05 ± 0.02	0.02 ± 0.03	0.01 ± 0.02	ND
2,4-DHBA	0.42 ± 0.08	2.07 ± 0.00	0.12 ± 0.16	0.30 ± 0.07	0.12 ± 0.04	ND	0.03 ± 0.04	0.01 ± 0.01	ND	0.76 ± 0.36	2.60 ± 0.00	0.98 ± 0.06
pCA	11.48 ± 3.10	19.79 ± 5.98	11.76 ± 1.66	298.47 ± 50.15	581.74 ± 21.79	554.45 ± 5.21	120.78 ± 15.74	117.67 ± 9.20	106.07 ± 47.90	209.83 ± 8.63	341.28 ± 19.74	284.05 ± 29.83
GA	0.02 ± 0.02	0.36 ± 0.02	0.45 ± 0.31	0.01 ± 0.01	ND	ND	0.01 ± 0.02	0.09 ± 0.12	ND	ND	ND	ND
HA	ND	ND	1.04 ± 0.19	ND	ND	ND	0.08 ± 0.11	ND	ND	ND	ND	ND
CA	7.94 ± 0.37	17.31 ± 0.12	23.38 ± 2.96	96.89 ± 5.16	127.87 ± 9.14	133.89 ± 7.81	252.30 ± 55.92	177.14 ± 24.65	156.17 ± 3.34	8.15 ± 0.38	22.96 ± 2.84	11.75 ± 4.87
3,4-DHCA	ND	ND	ND	ND	ND	ND	0.03 ± 0.04	0.02 ± 0.03	ND	0.05 ± 0.07	0.02 ± 0.02	0.01 ± 0.01
HVA	0.79 ± 1.12	15.79 ± 0.32	19.47 ± 2.79	100.20 ± 10.55	134.05 ± 3.52	123.71 ± 24.40	264.16 ± 63.75	173.96	146.22 ± 8.09	7.85 ± 0.10	29.23 ± 11.49	9.30 ± 13.15
SYRALD	0.20 ± 0.23	ND	ND	0.92 ± 0.14	2.30 ± 1.58	3.40 ± 0.02	0.06 ± 0.04	ND	ND	0.85 ± 0.08	0.06 ± 0.08	0.76 ± 1.07
FA	170.90 ± 8.50	232.81 ± 7.03	300.29 ± 56.19	880.57 ± 166.10	1500.71 ± 230.04	2038.59 ± 30.01	640.55 ± 35.17	1306.02 ± 120.10	828.01 ± 53.40	1797.34 ± 27.50	2884.67 ± 922.74	2023.40 ± 225.61
IFA	45.85 ± 0.11	55.81 ± 4.39	54.26 ± 14.21	74.69 ±.88	7.50 ± 1.02	49.71 ± 1.38	72.63 ± 10.06	8.33 ± 3.98	24.49 ± 2.81	124.92 ± 9.22	10.41 ± 1.75	29.50 ± 8.58
HFA	ND	ND	0.58 ± 0.82	0.04 ± 0.07	ND	ND	ND	ND	ND	0.01 ± 0.02	ND	ND
SYRA	17.52 ± 2.47	13.40 ± 2.92	19.80 ± 2.95	34.05 ± 0.95	74.21 ± 12.02	56.66 ± 8.31	8.52 ± 1.69	15.19 ± 1.86	8.98 ± 2.79	5.37 ± 1.40	42.49 ± 13.99	19.74 ± 7.14
SNA	11.05 ± 0.85	6.06 ± 0.34	4.79 ± 0.03	50.61 ± 7.23	45.83 ± 5.66	58.54 ± 5.99	12.53 ± 0.98	6.19 ± 1.75	3.63 ± 0.75	28.96 ± 7.44	91.58 ± 3.00	36.20 ± 15.77
TOTAL	281.40 ± 10.78	375.23 ± 8.25	444.62 ± 45.25	1631.24 ± 227.46	2530.83 ± 248.11	3077.71 ± 2.20	1464.17 ± 184.19	1863.37 ± 185.37	1317.51 ± 2.21	2202.51 ± 34.93	3445.04 ± 913.04	2433.33 ± 311.17

Raw samples were analysed in triplicate, cooked and digested samples in duplicate. All results are expressed as mean ± standard deviation (SD). ND = not detected.

**Table 3:** Cooking protocol for grains.

Cereal Grain	Initial Weight (g)	Volume of Water Used (mL)	Cooking Time (min)	Cooling Time (min)	Final Weight (g)
Red fonio	101.13	600	15	15	551.86
Red millet	100.29	550	36	15	178.92
Red sorghum	100.61	500	43	10	176.89
White corn	100.33	750	79	10	152.99

## Data Availability

Sequencing reads were deposited in the National Center for Biotechnology Information Sequence Read Archive (NCBI SRA; BioProject ID PRJNA993861).

## Supplementary Materials

The following supporting information can be downloaded at: https://www.mdpi.com/article/10.3390/ijms241814111/s1.
