# Supplementary material for: Cooking, Digestion, and In Vitro Colonic Fermentation of Nigerian Wholegrains Affect Phenolic Acid Metabolism and Gut Microbiota Composition
Source: Int J Mol Sci. 2023 Sep 14;24(18):14111. doi: 10.3390/ijms241814111 (PMC10531640; doi:10.3390/ijms241814111)
Supplement: Supplementary file 1 [file ijms-24-14111-s001.zip › table S1 submission.pdf]

Supplementary Table S1: LC-MS detection parameters of phenolic acids.

Phenolic acids	Ion mode	MW	Parent ion (m/z)	Fragments (m/z)	CV (V)	CE (V)	Cap(kV)
Vanillic acid	+	168.2	169	151,123*,108	30	12, 13, 16	2.4
Isovanillic acid	+	168.2	169.5	151,123*,108	30	12, 13, 16	2.4
4-hydroxybenzaldehyde	-	122.1	121.1	92*	25	20	2.4
3-hydroxybenzaldehyde	-	122.1	121.1	92*	25	17	2.4
4-hydroxybenzoic acid	-	138.1	137.1	108, 93* ,65	25	23,10,21	2.4
3-hydroxybenzioc acid	-	138.1	137.1	108, 93* ,65	25	23,10,21	2.4
Salicylic acid	-	138.1	137.1	93*, 65, 75	25	23,23,25	2.4
4-hydroxyphenylacetic acid	-	152.2	151.1	136, 107*, 92	25	12,10,19	2.4
3-hydroxyphenylacetic acid	-	152.2	151.2	136, 107*, 92	25	12,10,19	2.4
2-hydroxyphenylacetic acid	-	152.2	151.2	136, 107*, 92	25	12,10,19	2.4
Vanillin	-	152.1	151.1	136*, 107, 92	25	12,10,19	2.4
3,4 // 3,5 -dihydroxy benzoic acid sum	-	154.1	153.1	109*, 67, 65	25	10,16, 12	2.4
2,5-Dihydroxybenzoic acid	-	154.1	153.1	109*, 67, 65	25	10,16, 12	2.4
2,4-Dihydroxybenzoic acid	-	154.1	153.1	109*, 67, 65	25	10,16, 12	2.4
Gallic acid	-	170.1	169.1	151, 125*, 79	25	13,13,20	2.4
Homovanillic acid	-	182.2	181.7	137*, 121, 109, 59	25	10, 16, 17,17	2.4
Syringic acid	-	198.2	197.2	182*, 167, 121	25	13,17,20	2.4

p-Coumaric acid	-	164.2	163.2	119*, 117, 93, 91	25	13,22,27,20	2.4
Caffeic acid	-	180.2	179.2	135* ,107,79	25	15,25,23	2.4
Dihydrocaffeic acid	-	182.2	181.2	137, 121, 109 ,59*	25	10, 16, 17,17	2.4
Ferulic acid	-	194.2	193.2	178*,149,134,103	25	10,15,15,10	2.4
Isoferulic acid	-	194.2	193.2	178*,149,134,103	25	10,15,15,10	2.4
Hydroferulic acid	-	196.2	195.2	136*, 121, 119	25	15, 26,17	2.4
Sinapic acid	-	224.2	223.2	208*, 164, 149,120	25	12, 15, 19,27	2.4
Hippuric acid	-	179.2	178.2	134*, 77, 56	25	11, 14, 15	2.4
Syringaldehyde	-	182.2	181.2	166, 151* ,123	25	12, 18,25	2.4
