# Supplementary material for: Cooking, Digestion, and In Vitro Colonic Fermentation of Nigerian Wholegrains Affect Phenolic Acid Metabolism and Gut Microbiota Composition
Source: Int J Mol Sci. 2023 Sep 14;24(18):14111. doi: 10.3390/ijms241814111 (PMC10531640; doi:10.3390/ijms241814111)
Supplement: Supplementary file 1 [file ijms-24-14111-s001.zip › table S2 submission.pdf]

Supplementary Table S2: Calibration curve parameters for all phenolic acids, Limit of Detection (LOD) and Limit of Quantification (LOQ).

Compound name	Conc. range (μg/ml)	eq. slope	R^2 value	LOD (ng/ml)	LOQ (ng/ml)
Vanillic acid	0.05 - 100	2106.4	0.9977	0.07	0.24
Isovanillic acid	0.05 - 100	9236.9	0.9974	42.58	141.94
4-hydroxybenzaldehyde	0.05 - 100	2742.8	0.9966	2.43	8.09
3-hydroxybenzaldehyde	0.05 - 100	304.88	0.995	120.49	401.62
4-hydroxybenzoic acid	0.05 - 100	3807.9	0.9987	63.08	210.28
3-hydroxybenzioc acid	0.05 - 100	667.46	0.9642	23	76.6
Salicylic acid	0.05 - 100	9573.8	0.9974	2.24	7.46
4-hydroxyphenylacetic acid	0.05 - 100	1832.5	0.9967	23.66	78.88
3-hydroxyphenylacetic acid	0.05 - 100	9790.9	0.9984	1.79	5.96
2-hydroxyphenylacetic acid	0.05 - 100	9790.8	0.9984	4.65	15.51
Vanillin	0.05 - 100	1032	0.9954	662	2209
3,4 // 3,5 -dihydroxy benzoic acid sum	0.05 - 100	6022.5	0.9977	3.86	12.88
2,5-Dihydroxybenzoic acid	0.05 - 100	3521.3	0.9997	5.09	16.96
2,4-Dihydroxybenzoic acid	0.05 - 100	2757.6	0.9998	27.04	90.14
Gallic acid	0.05 - 100	2244.8	0.9958	7.06	23.53
Homovanillic acid	0.05 - 100	44.597	0.9973	755.73	2519.09
Syringic acid	0.05 - 100	54.869	0.9949	2.02	6.72
p-Coumaric acid	0.05 - 100	12192	0.997	3.02	10.05

Caffeic acid	0.05 - 100	7290.1	0.9972	9.43	31.44
Dihydrocaffeic acid	0.05 - 100	623.65	0.9949	1.62	5.4
Ferulic acid	0.05 - 100	993.63	0.9997	0.16	0.52
Isoferulic acid	0.05 - 100	73.939	0.9961	542.28	1807.59
Hydroferulic acid	0.05 - 100	1103.7	0.9999	40.81	136.03
Sinapic acid	0.05 - 100	1154.3	0.9999	0.11	0.37
Hippuric acid	0.05 - 100	2206.9	0.9955	5.15	17.15
Syringaldehyde	0.05 - 100	606.35	0.9944	479.25	1597.52
